# The Training Characteristics of World-Class Male Long-Distance Cross-Country Skiers

**DOI:** 10.3389/fspor.2021.641389

**Published:** 2021-02-25

**Authors:** Per-Øyvind Torvik, Guro Strøm Solli, Øyvind Sandbakk

**Affiliations:** ^1^Department of Sports Sciences and Physical Education, Nord University, Levanger, Norway; ^2^Department of Neuromedicine and Movement Science, Centre for Elite Sports Research, Faculty of Medicine and Health Sciences, Norwegian University of Science and Technology, Trondheim, Norway

**Keywords:** XC skiing, endurance training, strength training, speed training, double poling

## Abstract

**Purpose:** To investigate the training characteristics of world-class long-distance cross-country skiers.

**Methods:** Twelve world-class male long-distance cross-country skiing specialists reported training from their best season, through a questionnaire and follow-up interviews. Training data were systemized by training form (endurance, strength, and speed), intensity [low- (LIT), moderate- (MIT), and high-intensity training (HIT)], and exercise mode, followed by a division into different periodization phases. Specific sessions utilized in the various periodization phases were also analyzed.

**Results:** The annual training volume was 861 ± 90 h, consisting of 795 ± 88 h (92%) of endurance training, 53 ± 17 h (6%) of strength training, and 13 ± 14 h (2%) of speed training. A pyramidal (asymptotic) endurance training distribution was employed (i.e., 88.7% LIT, 6.4% MIT, and 4.8% HIT). Out of this, 50–60% of the endurance training was performed with double poling (DP), typically in the form of a daily 3- to 5-h session. A relatively evenly distributed week-to-week periodization of training load was commonly used in the general preparation period, whereas skiers varied between high-load training weeks and competition weeks, with half the training volume and a reduced amount of DP during the competition period.

**Conclusions:** To match the specific demands of long-distance cross-country skiing, specialized long-distance skiers perform relatively long but few training sessions and use a pyramidal intensity distribution pattern and a large amount of training spent using the DP technique.

## Introduction

Competitive cross-country (XC) skiing consists of two main types of event: (1) the Olympic disciplines, with competition formats ranging from short (~1.5 km) sprint competitions to 50-km-distance races performed in hilly terrain in the classical or skating styles, and (2) long-distance XC skiing with distances mainly ranging from 40 to 90 km performed in a more steady terrain and the majority of the races performed in the classical style.

While the Olympic disciplines include the use of and transition between many different subtechniques (Sandbakk and Holmberg, 2017; Solli et al., 2018, 2020), the relatively flat terrain profiles in long-distance events are often won on skis without grip wax, using solely the double-poling (DP) subtechnique (Sagelv et al., 2018; Zoppirolli et al., 2018, 2020; Skattebo et al., 2019; Stöggl et al., 2020) As a consequence of these demands and the increasing popularity of long-distance XC skiing, such as the Visma Ski Classics (VSC) series, long-distance XC skiers have fully specialized their training for performance in long-distance events (Skattebo et al., 2019). However, in contrast to the detailed examinations of physiological profiles and training characteristics of Olympic XC skiers (Sandbakk et al., 2011, 2016; Tønnessen et al., 2014; Sandbakk and Holmberg, 2017; Solli et al., 2017), these factors have been almost unexplored among skiers who have specialized in long-distance XC.

Extensive use of the DP subtechnique in long-distance races requires a well-developed DP technique, as well as upper-body strength and endurance capacity. A recent study compared the physiological capacities in DP between long-distance and Olympic XC skiers (Skattebo et al., 2019). While the study showed similar DP performances (i.e., time-to-exhaustion), long-distance skiers had lower peak oxygen uptake [maximal aerobic capacity (Vo_2peak_)] and better skiing efficiency (i.e., lower O_2_ cost) than Olympic skiers (Skattebo et al., 2019). Furthermore, Sagelv et al. (2018) reported that long-distance skiers performed better in DP than Olympic XC skiers and had lower blood lactate concentration, heart rate, and rating of perceived exertion at submaximal workloads due to higher DP efficiency. Accordingly, these findings may imply that the training of long-distance XC skiers, which includes more focus on DP than for Olympic XC skiers, leads to superior DP efficiency but lower Vo_2peak_.

The only scientific report on training in long-distance XC skiers is from the study of Skattebo et al. (2019), who reported an annual training volume of 775 h, distributed as 83% low-intensity (LIT), 3% moderate-intensity- (MIT), 6% high-intensity (HIT), 7% strength, and 2% speed training. This is within the range of training distribution reported for world-class Olympic XC skiers, with 750–950 h of annual training volumes distributed as 90–95% endurance training, 5–10% strength training, and 1–2% speed training (Sandbakk et al., 2011, 2016; Tønnessen et al., 2014; Solli et al., 2017). The endurance training intensity distribution observed in these elite Olympic XC skiers consisted of 88–91% LIT, 3–7% MIT, and 4–6% HIT, with an equal focus on classical and skating styles (Sandbakk et al., 2011, 2016; Losnegard and Hallén, 2014; Tønnessen et al., 2014; Solli et al., 2017). Although the training intensity distribution seems relatively similar, we would expect long-distance skiers to include more DP in their training, higher focus on flat terrain, and the inclusion of sport-specific sessions to meet the demands of long-distance XC skiing. However, such detailed information about the training characteristics of long-distance XC skiers is currently lacking. Although a recent study from Knechtle and Nikolaidis (2018) showed that ultramarathoners trained higher volumes at slower speeds than marathoners, detailed training data from long-distance and ultra-endurance athletes, including possible differences to the training of athletes competing in shorter events, are also lacking in other sports.

Therefore, the aim of this study was to investigate the training characteristics of world-class long-distance XC skiers, including detailed information about the distribution of training volume, intensity, and exercise modes, as well as specific session designs employed during the skiers' most successful season.

## Methods

### Participants

Twelve male Norwegian and Swedish long-distance XC specialists were recruited between May and September 2020. The inclusion criteria were as follows: (1) having competed in the VSC for at least 3 years, (2) having achieved at least two podium performances during their career, and (3) having achieved at least one podium performance during their most successful season. Participants reported that their best season occurred between 2010 and 2019 and thus reported the training from the nominated season. All skiers had progressively built up their training by using traditional Olympic XC ski training, as described by Sandbakk and Holmberg (2017). Five of the participants had retired as athletes, whereas seven were still competing at an elite level. The participants achieved a total of 154 podium performances in the VSC races (range per athlete: 2–27), and seven of these also had podium performances in the International Ski Federation World Cup. The other five did not reach a top national level in Olympic XC skiing before they started preparing for long-distance races.

The Regional Committee for Medical and Health Research Ethics, Trondheim, Norway, waives the requirement for ethical approval for studies of this type. Therefore, the ethics of the study were according to the institutional requirements, whereas approval for data security and handling was obtained from the Norwegian Center for Research Data. Prior to data collection, all participants provided written informed consent to voluntarily take part in the study. The participants were informed that they could withdraw from the study at any time without providing a reason for doing so. The characteristics of the participants are presented in Table 1.

**Table 1:** Anthropometric, physiological, and performance characteristics of 12 male long-distance cross-country skiers (mean ± SD) during their most successful season.

**Variable**	**Value**
Age, y	30.4 ± 3.7
Body height, cm	182.7 ± 5.6
Body mass, kg	77.0 ± 6.3
Body mass index, kg · m^−2^	23.1 ± 1.1
Maximum heart rate, beats · min^−1^	189 ± 8
Vo_2_max, L · min^−1^	6.2 ± 0.5
Vo_2_max, mL · min^−1^ · kg^−1^	80.1 ± 3.6
Total standing VSC	5.8 ± 7.5
FIS points (distance)^*^	24.5 ± 18.2
FIS points (sprint)^*^	55.3 ± 45.7

Vo_2max_, maximal aerobic capacity; VSC, Visma Ski Classics; FIS, International Ski Federation.

* *Lowest FIS points reported during the athlete's career*.

### Questionnaire

Data were collected via an online questionnaire (Nettskjema: https://nettskjema.no/) based on previous detailed training analyses of world-class XC skiers (Solli et al., 2017) and adjusted to the study aim by an expert panel of former athletes, coaches, a physiologist, and researchers with experience from similar projects. The questionnaire contained an introduction part including detailed description on how to answer the questionnaire, and each question was appropriately defined to avoid misinterpretation. To ensure that participants understood the questions, a pilot study with three participants was conducted before data collection commenced.

Designed to take 60–90 min to complete, the questionnaire contained 93 questions: zero closed-ended questions, 61 questions asking for a numeric value, one yes-or-no question, three multiple-choice questions, and 26 open-ended questions. Participants reported their demographic information, performance, and training characteristics during their most successful year in the VSC. The questionnaire also contained questions about their detailed design of typical training sessions used to meet the demands of the races in the VSC. During the data analysis, the participants were contacted to ensure compliance with the questionnaire responses and to verify the design and/or content of different training sessions.

All the recruited athletes completed the questionnaire, and their data were included in the final analysis. Ten of the athletes reported that their numeric data were collected from training diaries, while the remaining athletes reported training information from written notes. Because the questionnaire was in Norwegian, a translation process was performed to ensure validity when interpreting the questions in English.

### Annual Periodization

**Figure 1:**
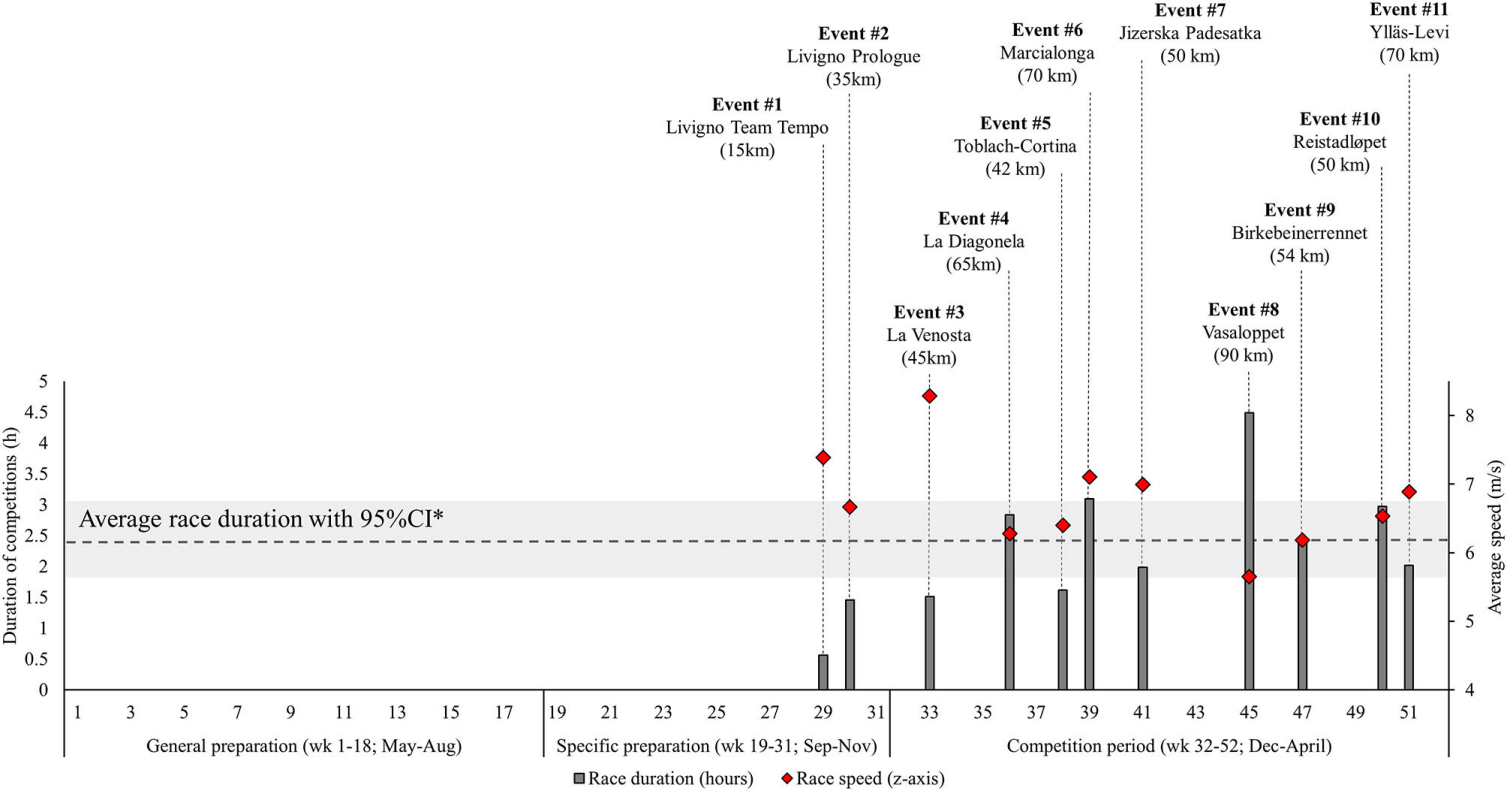
Illustration of the annual cycle in long-distance XC skiing with the timing of the competitions in the Visma Ski Classic 2019. Competition duration and speed are calculated based on the average for the three best skiers in each race and the course length reported in the official result lists (www.vismaskiclassics.com) of the 2018–2020 seasons. *The Livigno Team Tempo is excluded from the calculation of average race duration (dotted line) with 95% CI (gray area).

All training data were organized into periods [general preparation period (GP: May–August), specific preparation period (SP: September–November), and competition period (CP: December–April)], training form (endurance, strength, and speed), and intensity (LIT, MIT, and HIT). LIT refers to a training intensity below the first lactate threshold (LT^1^) (<2 mM blood lactate, 60–82% of maximal heart rate; HR_max_), MIT refers to an intensity between LT^1^ and LT^2^ (2–4 mM blood lactate, 82–87% of HR_max_), and HIT refers to an intensity above LT^2^ (>4 mM blood lactate, >87% of HR_max_) (Seiler and Kjerland, 2006). The participants used a combination of the session-goal approach and time in training zone to register training time, often called a modified session-goal approach as described in detail by Sylta et al. (2014). Strength training was categorized as heavy strength training and core stabilization (including muscular endurance). Speed training included maximal efforts of 10- to 20-s sprints or series of 10–15 plyometric jumps using ski specific movements. The use of exercise modes was categorized as being specific (DP on a ski ergometer or skiing/roller skiing) or other exercise modes. An overview of the annual cycle in long-distance XC skiing is presented in Figure 1.

### Statistics

Questionnaire responses were summarized in numerical values to facilitate statistical analysis.

Continuous variables are presented as mean ± SD and were examined for the assumption of normal distribution prior to analysis using a Shapiro-Wilk test, visual inspection of Q–Q plots, and histograms. Categorical variables are presented as absolute numbers and percentages. Data were processed and analyzed using IBM SPSS Statistics version 24 software for Windows (SPSS Inc., Chicago, IL, USA) and Office Excel 2016 (Microsoft Corporation, Redmond, WA, USA). A one-way repeated-measures analysis of variance was used for analyzing the differences in training across GP, SP, and CP. *Post hoc* comparisons were made using a Holm-Bonferroni correction. In cases where Mauchly test of sphericity indicated that the assumption of sphericity was violated, a Greenhouse–Geisser correction was performed. A *P* ≤ 0.05 was considered statistically significant. Effect size was evaluated with η^2^, where 0.01 < η^2^ <0.06 constitutes a small effect, 0.06 < η^2^ <0.14 constitutes a medium effect, and η^2^ > 0.14 constitutes a large effect (Cohen, 1988). To categorize free-text questions, two researchers performed independent content, frequency, and consistency analyses until consensus was reached. Direct verbatim quotations were used to inform interpretation. Descriptive data for continuous variables were recorded as means (SD), and for categorical variables as totals and percentages. For continuous variables, the Shapiro–Wilk test and standard visual inspection were used to examine the assumption of normality.

## Results

**Figure 2:**
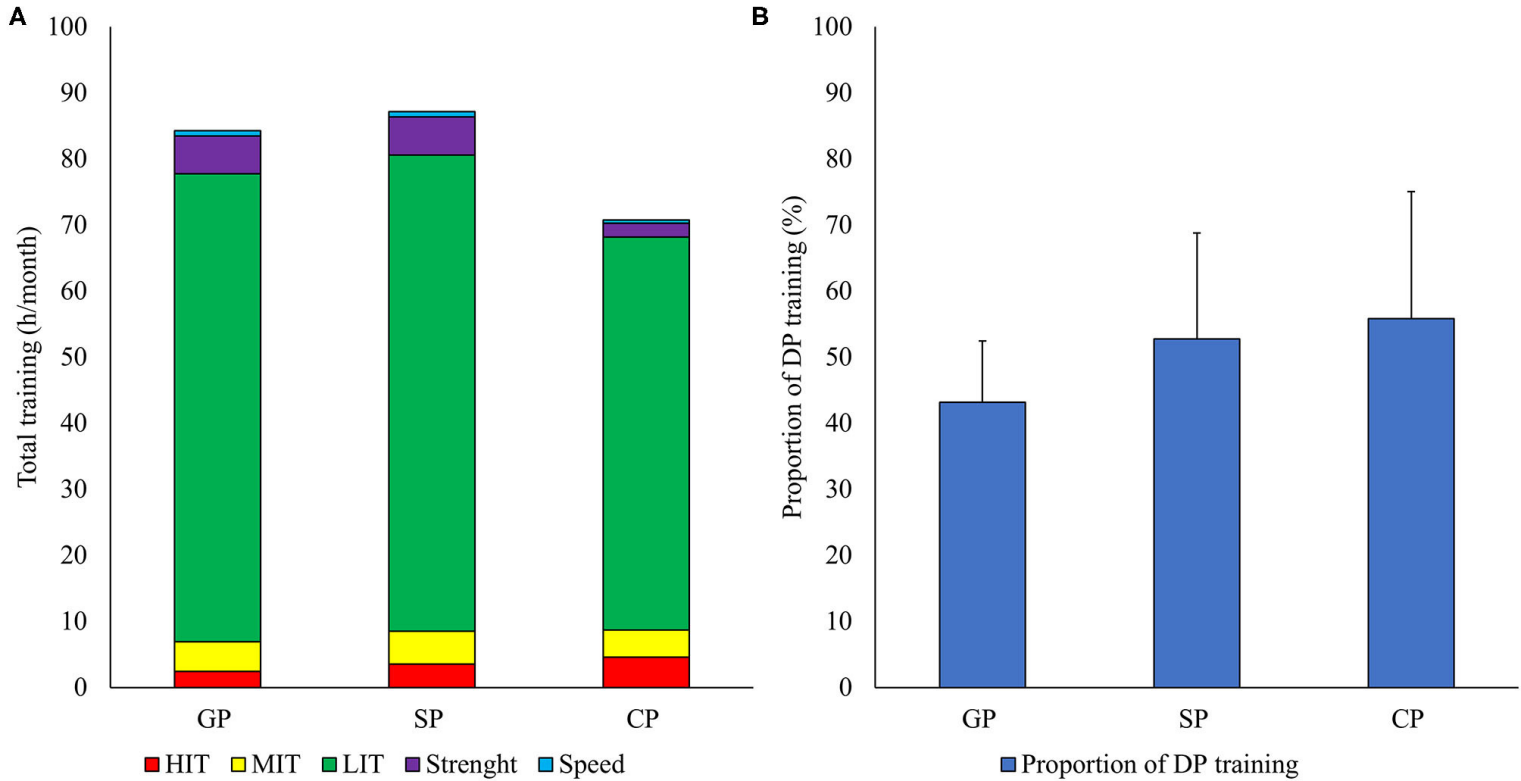
Training distribution **(A)** across the annual period [general preparation period (GP: May–August), specific preparation period (SP: September–November), and competition period (CP: December–April) during the athletes' most successful seasons distributed as endurance [low- [LIT], moderate- [MIT], and high-intensity [HIT]], strength, and speed training, as well as the proportion of training using the double poling (DP) subtechnique **(B)** for world-class long-distance cross-country skiers.

The annual training volume was 861 ± 90 h, consisting of 795 ± 88 h (92%) endurance training, 53 ± 17 h (6%) strength training, and 13 ± 14 h (2%) speed training. Periodical training patterns across the different period of the annual cycle are presented in Figure 2A. There was a change in training volume across period (GP: 85 h, SP: 87 h, and CP: 75 h; *P* = 0.017, η^2^ = 0.350) with the training volume being significantly higher in GP (*P* = 0.024) and SP (*P* = 0.011) compared to CP.

All athletes reported to normally use a traditional periodization model with a relatively even week-to-week distribution of training load (including the use of endurance and strength/speed training) during GP. Accordingly, their microcycle periodization was modest, and they kept training volumes high (i.e., 20–25 h/wk) for 2–3 weeks followed by a week with lower training load every 3–4 weeks or when they perceived a need for restitution. The competitive season for long-distance skiers lasts until late April, which requires a period of less training in May and beginning of June followed by a gradual progression of both training volume and intensity from June to September. Two of the athletes reported a strict microperiodization system consisting of 5/2 and 7/1 training/resting days. Five skiers reported some blocks with emphasis on specific qualities during the GP; three skiers included a couple of blocks of strength training, two had a few blocks of DP training, and one reported one to three blocks of increased amounts of HIT (5 HIT sessions in 6 days). All athletes reported more structured and accurate intensity control in SP than in GP. The same accurate intensity control was maintained during CP, but the training volume/load was determined by the competition schedule, with 10–12 h of weekly training in weeks with competitions and 20–30 h during weeks without competitions.

### Endurance Training

Annual LIT, MIT, and HIT volumes were 706 ± 92, 51 ± 24, and 39 ± 24 h, respectively, corresponding to an intensity distribution consisting of 88.7 ± 4.8% LIT, 6.4 ± 2.7% MIT, and 4.8 ± 2.8% HIT (including competitions). No significant differences between period were observed for LIT (GP: 71 h, SP: 72 h, and CP: 63 h; *P* = 0.071, η^2^ = 0.214) or MIT volume (GP: 4.5 h, SP: 5.0 h, and CP: 4.1 h; *P* = 0.034, η^2^ = 0.093). However, HIT volume (GP: 2.4 h, SP: 3.5 h, and CP: 4.6 h; *P* = 0.010, η^2^ = 0.342) was significantly higher in CP compared to GP (*P* = 0.017).

### Strength and Speed Training

The periodical volume of strength training was significantly different between the annual period (GP: 5.7 h, SP: 5.8 h, and CP: 2.1 h; *P* < 0.001, η^2^ = 0.590), with the strength volume being lower in CP compared to both GP (*P* < 0.001) and SP (*P* = 0.009). The proportion of heavy training vs. core stabilization training was relatively stable across GP (63 vs. 37%), SP (63 vs. 37%) and CP (65 vs. 35%). No difference in speed training was observed between the annual period (GP: 0.8 h, SP: 0.8 h, and CP: 0.5 h; *P* = 0.368, η^2^ = 0.087).

### Exercise Modes

A total of 48 ± 13% of endurance and speed training was conducted as DP (ski ergometer, roller skiing, and skiing). The amount of DP training (GP: 43% h, SP: 53%, and CP: 56% h; *P* = 0.013, η^2^ = 0.328) was significantly higher in SP (*P* = 0.049) and CP (*P* = 0.041) compared to GP (Figure 2B). Examples of the most used sport-specific training sessions and representative training weeks through the annual season is presented in Tables 2, 3.

**Table 2:** Sport-specific training methods for long-distance XC skiing, categorized as continuous, mixed, and interval-based training sessions.

	**Training method**	**Exercise modes**	**Description**
Continuous training	Long-distance specific	DP	3–8 h DP sessions, flat, and undulating terrain, with or without inclusion of sprints (10 × 10–12 s)
	Long-distance non-specific	All	2–4 h Low-intensive steady-state running, skating, or classic skiing on undulating terrain
	Long-distance mix of exercise modes	All	Low-intensive steady-state sessions changing the exercise mode in midsession. For instance, 2 h DP + 2 h running
	Progressive long distance	DP	Progressive session starting with 1.5–2.5 LIT followed by 0.5- to 1.15-h MIT and 0.5-h HIT Progressive 2- to 4-h session interspersed with sprints, and maximal effort during one uphill at the end of the session
	Competitions/tests or simulated competitions	DP Running	Competitions or test races ranging from 30 min to 2 h, often simulating the terrain of one of the main races during CP
Mixed training sessions	LIT + intervals	DP	2- to 5-h LIT followed by an interval session at moderate and/or high intensity. Typical sessions (5–6 × 5–6 min, 15 × 3 min, 30 × 1 min, 5 × 2 min, 45/15 s in 30 min)
	Interval + LIT	DP	Session started with an MIT or HIT interval (examples below), followed by 2- to 3-h LIT to simulate the fast start in races
	Strength + LIT	DP	2- to 4-h LIT before, during or after a strength session (heavy strength training + muscular endurance as described below)
Interval training sessions	MIT intervals	DP DIA Running	0.5- to 1-h warm-up followed by intervals at moderate intensity. Typical sessions: 4–6 × 8–15 min, 10 × 5–6 min, 15 × 3 min with 1–2 min recovery between intervals
	HIT intervals	DP Running DIA Running with poles	0.5- to 1-h warm-up followed by intervals at high intensity. Typical sessions: 4–6 × 4–6 min uphill, 5 × 10 min undulating terrain, 10 × 2–3 min with 2–3 min recovery between intervals. Short intervals such as 3–5 × 8–10 min (40/20 s, 45/15 s or 30/15-s work/rest with 2-min rest between intervals)
	Competition preparations	DP	0.5- to 1-h warm-up followed by intervals at high intensity often in easy terrain, to achieve high speed. Typical session: 4 × 6 min, 5–4–3–2–1 min, 3 × (3–2–1 min), with 1- to 3-min rest between intervals
	Lactate production training	DP	0.5- to 1-h warm-up followed by intervals at maximal effort. Typical sessions: 10–20 × 1 min with 1- to 3-min rest between intervals, 3 × (6 × 1 min), with 2-min rest between intervals, and 5-min rest between series
Strength & speed	Heavy strength		5 × 5 repetitions of 5–7 sets using exercises such as deadlift, squat, clean, pull-down, chins, toes to bar, back extension, pull over, dips, bench press (narrow grip)
	Muscular endurance		5–10 series of 6–12 repetitions with relatively short rest (1 min) between sets. Typical session: 10 × 10 repetitions of chins with start every minute or series of 1–2–3–4–5–6–7–8–9–10–9–8–7–6–5–4–3–2–1 repetitions, with start every minute
	Core stabilization		20–50 repetitions of different exercises targeting core stabilization or 45/15-s work/rest for 20–30 min using different exercises involving red core (slings), Olympic rings, elastic bands, and medicine balls
	Sprints	DP	10–15 × 10–15 s maximal effort, typically during long-distance LIT sessions with 2- to 3-min active recovery between sprints

*All, all exercise modes; DP, double poling; DIA, diagonal stride; LIT, low-intensity endurance training; MIT, moderate-intensity endurance training; HIT, high-intensity endurance training; CP, competition period*.

**Table 3:** Representative training week examples through the annual season for long-distance cross-country skiers.

**Day**	**General preparation period (GP)**	**Specific preparation period (SP)**	**Competition period (CP)^**†**^**
Mon	M: 3-h LIT long-distance DP^*^ E: 1- to 1.5-h LIT long-distance running	M: 3-h LIT long-distance DP, with sprints E: 1- to 1.5-h LIT long-distance running	M: 2-h LIT long-distance DP, with sprints E: Rest
Tue	M: 3.5-h LIT long-distance running E: Rest	M: 4-h LIT long-distance running E: Rest	M: 3-h LIT long-distance DP E: 1-h LIT long-distance running
Wed	M: 1-h LIT warm-up 1-h MIT intervals in DP^‡^ (5 × 10 min) 1-h LIT cool-down E: 1h Strength training: Core stabilization and heavy strength	M: 1-h LIT warm-up to 1.15-h MIT DP (Continuous) 1-h LIT cool-down E: Rest	M: 0.5-h LIT warm-up 1-h MIT intervals in DP (6 × 8 min) 0.5-h LIT cool-down E: 1-h Strength training: Core stabilization and muscular endurance
Thu	M: 2-h LIT long-distance running E: Rest	M: 4-h LIT long-distance DP followed by 1 h muscular endurance and heavy strength training E: Rest	M: 3-h LIT long-distance DP E: Rest
Fri	M: 3-h LIT long-distance DP, with sprints E: Rest	M: 2-h LIT long-distance DP, with sprints E: Rest	M: 3-h LIT long-distance mix of exercise modes (Classic, Skating and DP) E: Rest
Sat	M: 2-h LIT DP 0.6-h MIT intervals in DP (6 × 5 min) 0.5-h LIT cool-down E: 1 h Heavy strength training	M: 0.5-h LIT warm-up 0.8-h HIT intervals running with poles (7 × 4 min) 0.5-h LIT cool-down E: Rest	M: 0.5-h warm-up 0.8-h HIT intervals treadmill running (6 × 5 min)^§^ 0.5 h cool-down E: Rest
Sun	M: 2- to 3-h LIT running, with sprints E: Rest	M: 2–3-h LIT long-distance DP, with sprints E: Rest	M: 4-h LIT long-distance DP, with sprints E: Rest
Total	Volume: 15–24 h Sessions: 5–10 Distribution: 87/4/0 % LIT/MIT/HIT	Volume: 22–25 h Sessions: 5–10 Distribution: 88/3/3 % LIT/MIT/HIT	Volume: 17–25 h Sessions: 5–10 Distribution: LIT 87/5/4 % LIT/MIT/HIT

* The LIT sessions varied from athlete to athlete in GP, from 2 to 5 h, and the evening training depended on the duration of the morning training.

‡ The MIT intervals were organized in different ways throughout the year, from 8 to 15 min with 1- to 2-min break and lasting from 45 to 75 min.

† The training example is from a week without a Visma Ski Classic race.

§ *During SP and CP, the athletes increased intensity into the area between MIT and HIT to keep up their maximal aerobic power, and they used non-specific modes for arm recovery. M, morning training; E, evening training; DP, double pooling; LIT, low-intensity endurance training; MIT, moderate-intensity endurance training; HIT, high-intensity endurance training*.

## Discussion

This study investigated the training characteristics of world-class male long-distance XC skiers during their most successful season, including detailed information about training volume, intensity distribution, exercise modes, periodization, and session designs. The main findings were as follows: the average annual training volume was 861 ± 90 h, including 795 h (92%) of endurance training, 53 h (6%) of strength training, and 13 h (2%) of speed training. Here, a pyramidal (asymptotic) endurance training distribution was employed (i.e., 88.7% LIT, 6.4% MIT, and 4.8% HIT), with 50–60% of the endurance and speed training performed using DP. This training included many long-distance sessions, typically performed as a daily 3- to 5-h session. The week-to-week periodization of endurance training load was relatively evenly distributed in GP and SP, while all the skiers maintained a high training volume during training weeks in the CP but halved their volume and reduced the amount of DP during weeks with competitions.

The average annual training volume of 861 ± 90 h performed by the skiers in this study is in line with the 750–950 h previously observed in Olympic distance XC skiers (Sandbakk and Holmberg, 2014, 2017). Also, in line with Olympic XC skiers, more than 90% of this overall volume of the long-distance XC skiers was endurance training, with the intensity distributed in a pyramidal pattern (i.e., 89% LIT, 6% MIT, and 5% HIT) (Stöggl and Sperlich, 2015). As previously reported for all successful XC skiers (Sandbakk and Holmberg, 2017), the most training was LIT, which is considered to provide an important foundation for long-term endurance adaptations, by increasing tolerance for high volumes of training without being injured or overloaded, as well as complementing training at higher intensities (Laursen, 2010; Sandbakk and Holmberg, 2017). However, the pyramidal distribution is related to more MIT than previously reported in Olympic XC skiers, who normally show a more polarized intensity distribution (Sandbakk and Holmberg, 2014, 2017), probably due to differences in competition demands. Large endurance training volumes, with the majority performed as LIT, are common among endurance and ultra-endurance athletes across sports (Knechtle and Nikolaidis, 2018). While studies from a range of endurance sports show either polarized or pyramidal intensity distributions (Stöggl and Sperlich, 2015), the intensity distribution of long-distance or ultra-endurance is currently lacking in the literature.

Most long-distance races are performed with DP in relatively even terrain, which is similar to the demands of longer-duration MIT sessions performed with DP common among long-distance XC skiers. This is supported by the latest research (Stöggl et al., 2020), who found the mean race intensity to be 82% of maximal heart rate during a long-distance skiing event. The training routines of long-distance specialists consist of relatively high training volumes (i.e., >850 h per year) with a pyramidal endurance intensity.

The skiers studied here performed MIT sessions about once or twice per week during GP and SP, but mainly in competition-free weeks in CP, as recovery of the arms and upper body was prioritized before competitions. Specifically, these MIT sessions were performed using DP, either with long intervals (8–15 min) with short breaks (1–2 min) or as continuous 45- to 75-min sessions. Such sessions aim to delay the duration-related fatigue of long-distance races, which leads to reduced coordination, and this directly or indirectly affects the ability to maintain muscle power throughout the competition (Zoppirolli et al., 2018).

During CP, most of the reported HIT time came from competitions. However, many HIT sessions performed by the long-distance skiers during GP and SP were designed to simulate the competitive demands of certain important races. This concept is similar to Olympic XC skiing, where the specialized HIT sessions target the demands of either sprint or distance skiing disciplines (Sandbakk and Holmberg, 2017). An example highlighted in this study was a skier who focused mainly on Vasaloppet reported that he started many sessions with 1 h of MIT, followed by 2 h of LIT, before finishing with 30–40 min of HIT. Other skiers describe similar approaches prior to winning the Marcialonga where they finished the LIT training sessions with a 15-min HIT using DP on steep uphill terrain, simulating the 3-km final uphill finish in this particular race. Both examples show how the MIT/HIT session designs are guided by the competition demands.

In addition, regular HIT sessions were performed to increase participants' general aerobic capacity, such as short intervals from 45 s to 5 min with 15-s to 3-min recovery periods. Many of these sessions were in a non-specific training mode, such as diagonal stride, running, or running with poles to recover the arms while stimulating their Vo_2max_. In this context, all skiers studied had a history of training for Olympic XC skiing and had an average Vo_2max_ of ~80 mL · min^−1^ · kg^−1^ in their best year as a long-distance skier. The previous focus on Olympic XC skiing may have led to the development of a high maximal aerobic capacity (Holmberg, 2015) that can be maintained with smaller amounts of HIT when specializing in long-distance XC skiing, and greater focus on long-duration LIT and MIT sessions.

After reduced training load in May and beginning of June, a gradual progression of both training volume and intensity from June to September was present in these long-distance skiers. The week-to-week periodization of this training load, including the distribution of LIT, MIT, and HIT, was relatively evenly distributed in GP and SP, with an overall reduction of training volume during CP. Although a few skiers included a few blocks of strength training or focus on the DP mode, block periodization was not greatly used and not systematically as previously described for some seasons of the most successful female skier in history (Solli et al., 2017). In CP, all skiers had a pronounced periodization pattern, where high-volume training weeks were followed by competition weeks with half of the usual training load and less strain on the upper body to ensure muscular fitness for the competitions.

Accordingly, these long-distance skiers used a traditional periodization model, which emphasizes a mixed focus on training forms and intensities during all periods across the annual season, but with a progressive reduction in training volume substituted by higher training intensity and more specific training toward the CP (Matwejew, 1975; Tønnessen et al., 2014). Although several successful endurance athletes have organized their training according to this (Tønnessen et al., 2014, 2015; Rasdal et al., 2018; Solli et al., 2019), the model has received criticism because of the possible conflicting physiological responses produced by mixed training directed at many performance-related factors at the same time (Issurin, 2008). As an alternative, it has been suggested that a more effective way of organizing endurance training is to include defined blocks of increased focus on specific intensities, such as block periodization of HIT (Issurin, 2008).

More than 50% of the endurance training of the skiers was performed with DP, which is exceptionally high compared to Olympic XC skiers, who execute 50–60% of their endurance training in all specific exercise modes, including skiing in four to six subtechniques in both classical and skating styles (Sandbakk and Holmberg, 2017). In contrast, 50–60% of the endurance training of the skiers studied is in one specific subtechnique. This means that ~400–450 h per year were performed using DP, which is probably more than double the volume performed by Olympic XC skiers.

The high amount of DP in long-distance skiers may benefit their DP endurance capacity and technique in a wide range of different terrains and speeds. Consequently, Sagelv et al. (2018) and Skattebo et al. (2019) demonstrated better DP performance due to better DP efficiency, despite equal or lower Vo_2peak_, in long-distance skiers than in Olympic skiers. In addition, previous research has shown that O_2_ extraction in the upper body of XC skiers approaches that of the legs (Calbet et al., 2005). However, the high volume of upper-body training using the DP mode in long-distance XC skiers may allow them to extract more O_2_ from the upper body than shown in previous research on Olympic XC skiers.

Many of these DP sessions were relatively long (3–5 h) LIT sessions, and up to 8 h were reported by some skiers. In addition to being specific for the demands of long-distance XC skiing, these extended LIT sessions may provide a positive supplement to their previous training as Olympic XC skiers. Similar approaches have been used for decades by cyclists (Faria et al., 2005). However, there is a limit to the amount of DP a skier can tolerate, and as one skier in this study stated, “It is also a question of how much DP you can endure, without having motivational problems, injuries, or other setbacks.”

Giving priority to extended sessions requires longer recovery and often only one session each day. Consequently, fewer sessions are performed by long-distance XC specialists than by Olympic XC skiers, who normally have shorter sessions twice per day. The long-distance skiers who reported training two sessions per day used a shorter second session as active recovery, often in a non-specific training mode.

Strength and speed training are performed concurrently to this large amount of endurance training, and previous literature shows this to be beneficial for endurance performance through several mechanisms such as maintaining muscle mass, improving work economy/efficiency, and delaying fatigue during long-distance competitions (Sandbakk, 2018). However, only a certain level of strength is required, as one skier stated, “The goal of strength training is to become strong enough.” In this context, compared to previous data on Olympic skiers (Sandbakk and Holmberg, 2017), the total volume of strength training (6%, 53 h) reported was similar, whereas the amount of speed training (2%, 13 h) was lower.

Generally, the skiers placed strength training sessions in their schedule based on the goal of the session, e.g., strength training performed after a LIT session aimed to fatigue the upper body muscles with long-term LIT before mobilizing the specific muscles with strength exercises. Other sessions were aimed at developing movement-specific power and therefore took place directly after warm-up. As for types of strength exercises, the athletes agreed that upper-body and core exercises aimed at developing power in the DP movement were most important, with chins as an example of an exercise used by all athletes. Block periodization of strength training was only reported only by a few athletes, but several pointed out the importance of building up their strength early in the cycle to become “strong enough” to tolerate all the DP without getting injured.

Sessions mainly focused on speed training were not prioritized by long-distance specialists, but all participants reported having regularly included 5–10 short sprints in their LIT sessions. Such sessions target their ability to accelerate and maintain high speed during attacks or when they were in position to fight for victory at the end of a race. Therefore, these sessions are often performed at the end of LIT sessions. In addition, most of the best “sprinters” in this study had participated in sprint skiing events and could profit from their previous training in sprinting. Their training data and self-reported sprint ability might suggest that long-distance skiers' training routines have an unused potential to further develop their speed systematically.

### Methodological Considerations

The strength of this study is the high number of top-level long-distance XC skiers providing novel data on training associated with success in this sport. However, the study also has some limitations: (1) recall bias is a limitation of retrospective questionnaires; (2) we were unfortunately not able to recruit any female participants and thus to investigate potential sex differences and generalize the findings to the female population; and (3) as the authors used their own network in the recruitment process, potential selection bias such as including only Norwegian and Swedish skiers may have affected the findings.

## Conclusions

The training of world-class long-distance XC skiers consists of high volumes (i.e., 861 ± 90 h annually) where low-intensity endurance training predominates. More specifically, long-distance skiers perform relatively long but few sessions (i.e., regular 3- to 5-h sessions), use a pyramidal intensity distribution pattern (i.e., 88.7% LIT, 6.4% MIT, and 4.8% HIT), and spend much (50–60%) of their training time using the DP technique. In addition, competition-specific sessions, such as long-duration LIT-to-MIT finalized with HIT or sprint training, are specific features of the training of long-distance XC skiers. Accordingly, the training routines seem to match the specific demands of long-distance XC skiing, with competitions commonly performed as long-duration DP. The week-to-week periodization included relatively evenly distributed training loads in GP and SP. However, all skiers had a pronounced periodization pattern during CP, where high-volume training weeks were followed by competition weeks with half of the training load and less strain on the upper body to ensure muscular fitness for the competitions.

## Data Availability Statement

The raw data supporting the conclusions of this article will be made available by the authors, without undue reservation.

## Ethics Statement

The studies involving human participants were reviewed and approved by The Regional Committee for Medical and Health Research Ethics waives the requirement for ethical approval for studies of this type. Therefore, the ethics of the study were according to the institutional requirements, while approval for data security and handling was obtained from the Norwegian Center for Research Data. The patients/participants provided their written informed consent to participate in this study.

## Author Contributions

P-ØT and ØS planed and designed the study. P-ØT and GS performed the data collection. P-ØT, GS, and ØS analyzed and presented the data, authored and finalized the manuscript for publication, and have approved the final manuscript. All authors contributed to the article and approved the submitted version.

## Conflict of Interest

The authors declare that the research was conducted in the absence of any commercial or financial relationships that could be construed as a potential conflict of interest.

## Abbreviations

CP: Competition period
GP: General preparation period
HIT: High-intensity training
MIT: Moderate-intensity training
LIT: Low-intensity training
SP: Specific preparation period
VSC: Visma Ski Classics
XC: Cross country.
